# Humanized Major Histocompatibility Complex Transgenic Mouse Model Can Play a Potent Role in SARS-CoV-2 Human Leukocyte Antigen-Restricted T Cell Epitope Screening

**DOI:** 10.3390/vaccines13040416

**Published:** 2025-04-15

**Authors:** Jiejie Zhang, Feimin Fang, Yue Zhang, Xuelian Han, Yuan Wang, Qi Yin, Keyu Sun, Haisheng Zhou, Hanxiong Qin, Dongmei Zhao, Wanbo Tai, Jun Zhang, Zhang Zhang, Tiantian Yang, Yuwei Wei, Shuai Zhang, Shuai Li, Min Li, Guangyu Zhao

**Affiliations:** 1 School of Basic Medical Sciences, Anhui Medical University, Hefei 230032, China; zhangjiejie202202@163.com (J.Z.); fgh66666@163.com (F.F.); haishengs@ahmu.edu.cn (H.Z.); 2 State Key Laboratory of Pathogen and Biosecurity, Academy of Military Medical Sciences, Beijing 100071, China; hanxuelian@bmi.ac.cn (X.H.); wangyuan1@bmi.an.cn (Y.W.); yinqi@bmi.ac.cn (Q.Y.); s1010457183@163.com (K.S.); ytt_tjutcm@163.com (T.Y.); weiyuwei9872@163.com (Y.W.); shuaiz2023@163.com (S.Z.); 3 Laboratory of Advanced Biotechnology, Academy of Military Medical Sciences, Beijing 100071, China; zhangyue4381@126.com (Y.Z.); zhangjun@bmi.ac.cn (J.Z.); zzhnng@126.com (Z.Z.); 4 Public Health School, Mudanjiang Medical University, Mudanjiang 157011, China; 5 Changchun Institute of Biological Products Co., Ltd., Changchun 130012, China; qinhanxing2000@163.com (H.Q.); 15754306108@163.com (D.Z.); 6 Shenzhen Bay Laboratory, Institute of Infectious Diseases, Shenzhen 518132, China; taiwb@szbl.ac.cn; 7 School of Life Sciences, Anhui Agricultural University, Hefei 230036, China

**Keywords:** SARS-CoV-2, mouse model, human leukocyte antigen (HLA) complex, epitope, immunological evaluation

## Abstract

**Background:** COVID-19, caused by SARS-CoV-2, poses a significant threat to human health. Vaccines designed for T-cell epitopes play an important role in eliminating the virus. However, T cell epitope screening often requires the use of a large number of peripheral blood mononuclear cells (PBMCs) from infected or convalescent patients, and if MHC humanized mice can be used for epitope screening, they will not have to wait for enough PBMCs to be available to screen for epitopes, thus buying time for epitope confirmation and vaccine design. **Methods**: In this study, we used SARS-CoV-2 BA.5 to infect HLA-A11/DR1, C57BL/6, hACE2 mice, and detected body weight changes, viral load, and pathological changes after infection. Fourteen days after the HLA-A11/DR1 and C57BL/6 mice were immunized against inactivated viruses, IgG antibodies were detected in mouse serum using ELISA, and IFN-γ produced by peptide stimulation of splenocytes was detected by ELISpot. **Results**: There is no obvious pathogenic phenotype of SARS-CoV-2 infection in HLA-A11/DR1 mice. Specific IgG antibodies were detected in serum after immunization of inactivated virus in both HLA-A11/DR1 and C57BL/6 mice, but specific IFN-γ was detected in splenocytes of HLA-A11/DR1 mice. **Conclusions**: Although HLA-A11/DR1 mice are unable to replicate the virus effectively in vivo, they are able to generate cellular immune responses after immunization inactivated viruses. Therefore, it can be used as a tool to substitute for human PBMCs in epitope screening, thus shortening the timeliness of T cell epitope screening and obtaining the immunogenicity information of new epitopes in a timely manner.

## 1. Introduction

Novel coronavirus infection, caused by severe acute respiratory syndrome coronavirus 2 (SARS-CoV-2), is an acute infectious disease with high infectivity [1]. SARS-CoV-2 can cause severe respiratory illnesses [2]. Since its emergence in 2019, the virus has rapidly spread worldwide, posing a significant threat to human health. Ongoing mutations continue to amplify its potential risks [3,4,5]. Therefore, researchers worldwide are striving to develop vaccines and drugs for the prevention and treatment of the novel coronavirus.

Animal models are essential for developing and preclinically evaluating vaccines and treatments [6,7]. Mice are commonly used in research due to their small size, short breeding cycle, and ease of experimental manipulation. They serve as valuable preclinical models for studying systemic diseases and viral infections, and for evaluating therapeutic interventions [8,9,10]. C57BL/6 mice are commonly used wild-type mice. The K18-hACE2 mice constructed by modifying the ACE2 receptor of mice are widely used in the research on the pathogenicity of the novel coronavirus. In addition, transgenic mice constructed by injecting human hematopoietic stem cells are also included. These mouse models have been well applied in the research on the novel coronavirus [11,12,13]. The Major Histocompatibility Complex (MHC) is a multigene family that encodes proteins essential for the adaptive immune response. In humans, the MHC is known as the human leukocyte antigen (HLA) [14,15]. Studies have shown that the genes and genetic materials of mice and humans have a high degree of similarity [16]. The major histocompatibility complex (MHC) is involved in encoding proteins and presenting antigens to T cells, playing an important role in both the adaptive and innate immune systems. Although the structure and function of MHCs are very similar between mice and humans, there are species differences in MHC restriction between them. The different functions of MHC restriction cause fundamental differences in the immune response status between mice and humans. Conventional mouse models are limited by the differences in antigen presentation and epitope selection between the MHC of mice and humans [17,18]. For this reason, ordinary wild-type mice cannot truly reflect the immune response status of the human body, especially the cellular immune status. Therefore, there is an urgent need for a mouse model that can simulate the human immune response [19].

The human immune response includes humoral immune responses and cellular immune responses, which work together to exert their effects. T cell epitopes are essential in immunity, with CD4+ T cells regulating immune responses and CD8+ T cells eliminating infected cells. In terms of immunological principles, the elimination of virus-infected cells in the body mainly depends on the targeted killing mediated by cytotoxic T lymphocytes (CTLs) [20]. Studies have shown that the T cell immune response induced by the COVID-19 vaccine can last for at least six months, which is of great significance for long-term protection. And since the T cell epitopes used in vaccine preparation are conserved, they can better cope with the impact caused by the continuous variation of SARS-CoV-2 [21]. Screening and identifying effective T cell epitopes is very important. Screening T cell epitopes needs to use human PBMCs to better reflect the immune response status of the human body, which requires a certain number of infected or convalescent patients to conduct experiments. This means that, in the event of a sudden emerging infectious disease, a large number of samples cannot be obtained in time for T cell screening, so there is a certain lag. If a tool could be used at the initial stage of infection to replace human PBMCs to screen out effective T cell epitopes, it would be very meaningful for vaccine design.

In this paper, we used a humanized mouse model HLA-A11/DR1 that can simulate the human immune response. It is a human HLA transgenic mouse (HLA-A11+/+/DR01+/+/H-2-β2m−/−/IAβ−/−). The preparation process and immunological functions of HLA-A11/DR1 mice have been reported in previous studies. By silencing the H-2-β2m and IAβ genes, the H-2 restriction function of the mouse itself is inhibited, and the endogenous MHC restriction (H-2) of the mouse is removed. Through the use of HLA-A11 promoter, light chain β2m, heavy chain α1 and α2 functional regions, α3 functional region, and transmembrane region of H-2-Db, the expression of human β2m and α1, α2 of HLA-A11 is achieved, forming an antigen binding groove with HLA-I restriction. The expression of HLA-II molecules is achieved by inserting HLA-DRA and HLA-DRB1*01:01 genes, so that the mouse acquires HLA-II restriction. This mouse model, which removes the endogenous MHC restriction of mice and has both HLA-I and HLA-II restrictions, can make the immune system of mice completely regulated by human MHC (HLA) during virus infection, so as to better simulate the human immune response status [22,23,24]. We used this humanized mouse model to verify its applicability in human epitope identification. Mice were immunized with inactivated virus and stimulated with HLA-restricted epitopes identified in previous reports and validated using human PBMCs. We observed that the model elicited a cellular immune response consistent with the human immune response, suggesting its potential for T cell epitope screening and enhanced responsiveness to emerging infectious disease outbreaks.

## 2. Materials and Methods

### 2.1. Ethical Statement

All animal experiment protocols were reviewed and approved by Institutional Animal Care and Use Committee of Academy of Military Medical Sciences (Permit Number: IACUC-IME-2022-004), and Changchun Institute of Biological Products Co., Ltd. (Changchun, China). (CCIBP 202406-04) in accordance with the relevant guidelines for the protection of animal subjects. All infectious experiments followed the standard operating procedures of the approved biosafety level-3 facility at Changchun Institute of Biological Products Co., Ltd.

### 2.2. Viruses and Animals

The SARS-CoV-2 BA.5 strain we used was isolated and preserved by the Changchun Institute of Biological Products Co., Ltd. (Changchun, China). Inactivated virus were preserved by the Laboratory of Advanced Biotechnology, Academy of Military Medical Sciences (Beijing, China). In our study, we used 6–8-week-old female HLA-A11/DR1 mice (18–20 g) to develop a humanized model. These transgenic mice (HLA-A11+/+/DR01+/+/H-2-β2m−/−/IAβ−/−) were used to model human immune responses [23]. Additionally, 6–8-week-old specific pathogen free (SPF)-grade female C57BL/6 mice weighing 18–20 g were purchased from SPF Biotechnology Co., Ltd. (Beijing, China). The 6–8-week-old female K18-hACE2 transgenic mice, also weighing 18–20 g, were obtained from GemPharmatech Co., Ltd. (Jiangsu, China).

### 2.3. SARS-CoV-2 Infection in Mice Experiment

The mice were divided into three main groups: HLA-A11/DR1, C57BL/6, and hACE2, with eight mice per group. Each main group was then randomly divided into two subgroups of four mice each. A total of 24 mice were intranasally infected with 1 × 10^4^ TCID_50_ SARS-CoV-2 (BA.5 strain), which is a lethal dose.

In each experimental group, the first subgroup was weighed every other day for 14 days to observe body weight changes following SARS-CoV-2 infection, with euthanasia performed on day 14. The second subgroup was euthanized on day 3 post-infection, and lung tissues were collected. Part of the lung was fixed in 4% paraformaldehyde for histopathological examination and SARS-CoV-2 nucleocapsid protein detection of the novel coronavirus on the lung tissue surface, while the remainder was placed in 1 mL TRIzol for RNA extraction and viral load measurement. Studies have shown that mice reach the peak of virus replication on the 3rd day after SARS-CoV-2 infection, and enter the recovery period 14 days after infection. Therefore, we selected these two time points to examine the pathogenic phenotype of mice after infection [25].

### 2.4. Extraction of Viral RNA and qRT-PCR

Viral loads in mouse lung tissues were measured using real-time quantitative PCR (qRT-PCR). RNA was extracted using the TRIzol method, reverse-transcribed into cDNA, and qRT-PCR was conducted in a 25 μL reaction system containing Premix Ex Taq, primers, probes, and cDNA. A system in which water is added in an amount equal to that of the cDNA was used as a negative control. The primers and probe used were EsgRNA-F (5′-CGATCTCTTGTAGATCTGTTCTC-3′), EsgRNA-R (5′-ATATTGCAGCAGTACGCACACA-3′), and EsgRNA-P3 (5′-ACACTAGCCATCCTTACTGCGCTTCG-3′).

### 2.5. Histopathological Analysis

After euthanizing the mice, on dpi 3, lung tissues were fixed in 4% paraformaldehyde, embedded in paraffin, sectioned, and stained with hematoxylin and eosin for histopathological examination. Pathological changes were evaluated using an optical microscope.

### 2.6. Immunohistochemistry

At dpi 3 post-SARS-CoV-2 infection, mouse lung tissues were collected, fixed in 4% paraformaldehyde, embedded in paraffin, sectioned, and the SARS-CoV-2 Nucleocapsid protein (Abcam, Cambridge, UK) monoclonal antibody was diluted at a ratio of 1:4000 for staining to detect its expression in the lung tissues of infected mice [26].

### 2.7. Immunization of Mice with SARS-CoV-2 Inactivated Virus

Four female HLA-A11/DR1 mice (aged 6–8 weeks) and four C57BL/6 mice (aged 6–8 weeks) were divided into two groups. SARS-CoV-2 was inactivated with β-propiolactone to obtain the inactivated virus. Each mouse was immunized with 3 × 10^3^ TCID_50_ inactivated SARS-CoV-2 wild-type strain through intramuscular injection. Fourteen days post-vaccination, blood and spleens were collected. Serum was used to detect specific antibodies, and spleens were placed in RPMI 1640 medium for detect IFN-γ production.

### 2.8. Screen HLA-Restricted Positive Epitope Peptides

HLA-A*11:01 and HLA-DRB1*01:01 restricted epitopes were selected from IEDB website to verify the cellular immune response function of HLA-A11/DR1 transgenic mice. These selected epitopes are conserved, and these epitope peptides were verified to be immunogenic through experiments such as MHC-peptide tetramer staining and intracellular cytokine staining (ICS) using human PBMCs samples, as reported in the literature.

### 2.9. ELISA

Each well of a 96-well plate was coated with 100 μL of SARS-CoV-2 S1 protein (2 μg/mL), sealed, and incubated overnight at 4 °C. The plate was then left at room temperature for 30 min to allow it to return to room temperature. The liquid in the wells was discarded, and after washing with 1× PBST, 200 μL of blocking buffer (2% BSA) was added to each well and incubated at 37 °C for 2 h. The liquid in the wells was discarded again, and after washing with 1× PBST, 100 μL of the test serum diluted in series was added to each well, with two replicate wells set up for each mouse. The serum was diluted starting from 1:10, with a 2-fold gradient dilution, resulting in a total of 11 dilution gradients. The plate was incubated at 37 °C for 1 h. After incubation, the liquid in the wells was discarded, the plate was washed with 1× PBST, and HRP-labeled goat anti-mouse antibody was added, followed by incubation at 37 °C for 30 min. After washing with 1× PBST, 100 μL of 3,3′,5,5′-Tetramethylbenzidine (TMB) two-component chromogenic substrate was added to each well, and the plate was incubated in the dark at 37 °C for 30 min. Then, 100 μL of ELISA stop solution was added to terminate the reaction. The optical density (OD) values at wavelengths of 450 nm and 630 nm were measured using a multifunctional microplate reader. The antibody titer in mice was determined with an OD value of 0.1 as the cutoff value.

### 2.10. ELISpot

We used the ELISpot plus: mouse IFN-γ (HRP) detection kit (Mabtech, Nacka, Sweden, product code: 3321-4HPW-10) to detect the IFN-γ factor secreted by mice after being immunized with the inactivated SARS-CoV-2 virus. The ELISpot 96-well plates were blocked with 200 μL RPMI 1640 medium containing 10% FBS and incubated at room temperature for 2 h. Four HLA-A11/DR1 mice and four C57BL/6 mice were euthanized 14 days after being immunized with the inactivated virus. Their spleens were collected to prepare single-cell suspensions. Each mouse had two replicate wells. The peptides we used were synthesized by SBS Genetech Co., Ltd. (Beijing, China)., which were provided by dissolving in 100% dimethylsulfoxide (DMSO) at 20 mg/mL, and stored at −20 °C before we used. For the experimental wells, 100 μL of HLA-restricted peptide with a concentration of 4 μg/mL (diluted in RPMI 1640 medium, Gibco, Grand Island, NY, USA) was added to each well. For the positive control group, concanavalin A (conA) was added, and for the non-stimulated group, 100 μL of RPMI 1640 medium was added. Each well was then supplemented with 100 μL of splenocytes at a concentration of 5 × 10^5^ cells per well. The plates were incubated in a cell culture incubator at 37 °C with 5% CO_2_ for 24 h. The IFN-γ produced by mouse spleen cells was captured by the ELISpot (Apex 1.1) PVDF membrane coated with anti-mouse IFN-γ. After incubation, the liquid in the wells was discarded, and the plates were washed with PBS. Then, 100 μL rat-derived anti-mouse IFN-γ monoclonal antibody (code: 3321-6) was added to each well, and incubated at room temperature for 2 h. The liquid in the wells was discarded again, and the plates were washed with PBS before adding streptavidin-HRP and incubating at room temperature for 1 h. The liquid in the wells was discarded once more, and the plates were washed with PBS. Then, 100 μL TMB substrate solution was added to each well, and the plates were incubated at room temperature in the dark for 2–15 min, and spots appeared on the PVDF membrane. The liquid in the wells was discarded, and the color development reaction was stopped by rinsing 2–3 times with deionized water. The plates were air-dried at room temperature and stored away from light for counting. The study was analyzed by the Mabtech IRIS FluoroSpot/ELISpot reader, using RAWspot technology (Nacka, Sweden) for multiplexing at the single-cell level.

### 2.11. Statistical Analyses

In this experiment, we used one-way analysis of variance (ANOVA) or *t*-tests to determine statistical significance. All statistical analyses were performed using GraphPad Prism 8.0.2.

In the pathogenic study of mice after SARS-CoV-2 infection, we used one-way ANOVA to compare the statistical differences in body weight change and viral load among HLA-A11/DR1, C57BL/6, and hACE2 mice after virus infection. The t-test was used to analyze the statistical differences in antibody production between mice that were not vaccinated with inactivated virus and those that were vaccinated with inactivated virus. The *t*-test was also used to analyze the statistical differences in the number of spots produced by each peptide stimulation in HLA-A11/DR1 and C57BL/6 mice after vaccination with inactivated virus.

## 3. Results

### 3.1. Weight Changes and Viral Replication in HLA-A11/DR1 Mice After SARS-CoV-2 Infection

**Figure 1:**
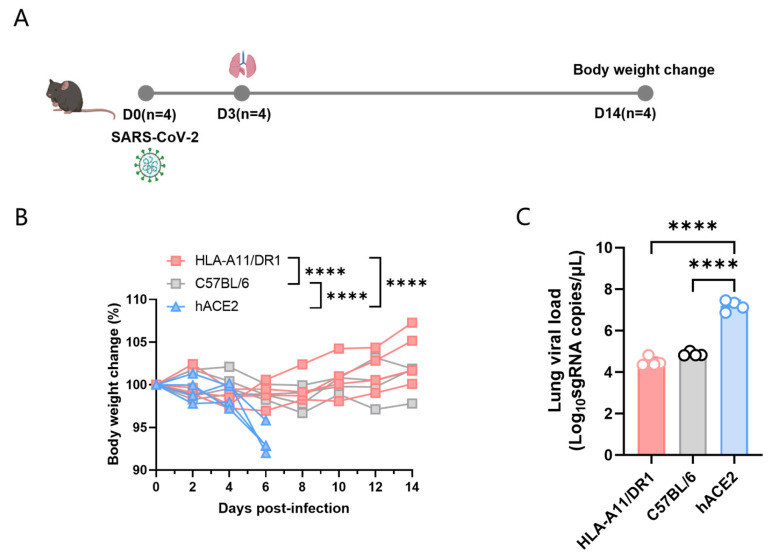
Weight changes and viral load in HLA-A11/DR1 mice infected with SARS-CoV-2. Mice were divided into three groups—HLA-A11/DR1, C57BL/6, and hACE2—and intranasally infected with 1 × 10^4^ TCID_50_ SARS-CoV-2 (BA.5 strain). Mice were weighed every 2 days over 14 days, and weight changes were expressed as percentages. On dpi 3, lung tissues were collected: the lung for histopathology, SARS-CoV-2 nucleocapsid protein detection and for viral load detection. (**A**) Weight changes in the three mouse groups after intranasal inoculation with the BA.5 virus strain. (**B**) Viral load detection in lung tissues of the three groups post-infection. (**C**) **** *p* < 0.0001.

To study the pathogenic characteristics of HLA-A11/DR1 mice after SARS-CoV-2 infection, we divided the experiment into three major groups, namely HLA-A11/DR1, C57BL/6, and hACE2 mice. We used wild-type C57BL/6 mice as negative controls, and hACE2 mice, which have been widely used in SARS-CoV-2 pathogenic studies, as positive controls. The mice were intranasally infected with 1 × 10^4^ TCID_50_ SARS-CoV-2 (BA.5 strain), and weighed every other day for 14 consecutive days (Figure 1A). The results showed that hACE2 mice, which are capable of being infected by SARS-CoV-2, exhibited a significant weight loss, while HLA-A11/DR1 mice and C57BL/6 mice showed almost no significant weight changes (Figure 1B).

To better detect the replication of SARS-CoV-2 in mice, we took lung tissue from the mice on day 3 post-infection, extracted tissue RNA using the TRIzol method, and then used qRT-PCR to determine the viral load in lung tissues. In this experiment, each group included four mice. The results showed that hACE2 mice could effectively replicate SARS-CoV-2 in vivo, while the viral loads in HLA-A11/DR1 and C57BL/6 mice were significantly lower than those in hACE2 mice, and there was no significant difference in viral loads between HLA-A11/DR1 and C57BL/6 mice (Figure 1C).

Overall, compared to hACE2 mice, HLA-A11/DR1 and C57BL/6 mice did not exhibit significant weight loss after SARS-CoV-2 infection, and the viral loads in these two groups of mice were significantly lower than those in hACE2 mice. These results indicate that HLA-A11/DR1 mice cannot effectively replicate SARS-CoV-2.

### 3.2. Lung Tissue Pathological Changes and Nucleocapsid Protein Distribution in HLA-A11/DR1 Mice After SARS-CoV-2 Infection

**Figure 2:**
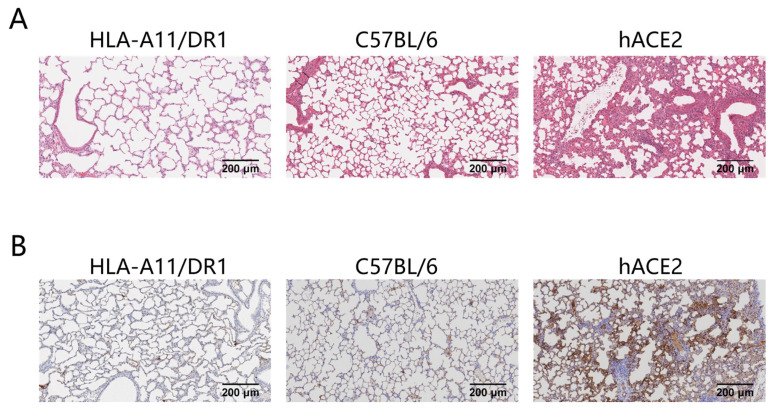
Histological and viral antigen detection in mouse lung tissues after SARS-CoV-2 infection. Mice were euthanized on dpi 3. The lung was fixed in 4% paraformaldehyde, paraffin-embedded, sectioned, and stained with H&E for pathological scoring. Lung tissue histology (original magnification: 10×, scale bar: 200 μm) (**A**). Distribution of SARS-CoV-2 nucleocapsid protein on lung tissue surfaces (original magnification: 10×, scale bar: 200 μm) (**B**).

Severe pneumonia can occur after SARS-CoV-2 infection. To study the pathological changes in lung tissue of HLA-A11/DR1 transgenic mice after SARS-CoV-2 infection, we fixed and sectioned the lung tissues of mice at 3 days post-infection (dpi) for histopathological examination. The research results showed that in hACE2 mice, large areas of consolidation in the lungs, an increase in lymphoid mononuclear cells, multifocal inflammatory cell infiltration in the alveoli, and partial formation of sheet lesions were observed, indicating parenchymal pathology in lung tissues. These findings were consistent with the previously detected high viral loads in lung tissues. In contrast, the lung tissue lesions in HLA-A11/DR1 mice and C57BL/6 mice were very mild or almost non-existent, suggesting that SARS-CoV-2 infection does not cause parenchymal pathology in the lungs of HLA-A11/DR1 mice (Figure 2A).

We also examined the distribution of viral antigens on the surface of lung tissues after mice were infected with SARS-CoV-2. The research results showed that the distribution of the SARS-CoV-2 nucleocapsid protein was detected in the lung tissues of hACE2 mice after infection, while no viral antigens were detected in the lung tissues of HLA-A11/DR1 mice and C57BL/6 mice (Figure 2B). In view of the above results, HLA-A11/DR1 transgenic mice cannot effectively replicate SARS-CoV-2 in vivo.

### 3.3. HLA-A11/DR1 Mice Can Generate Humoral Immune Responses

**Figure 3:**
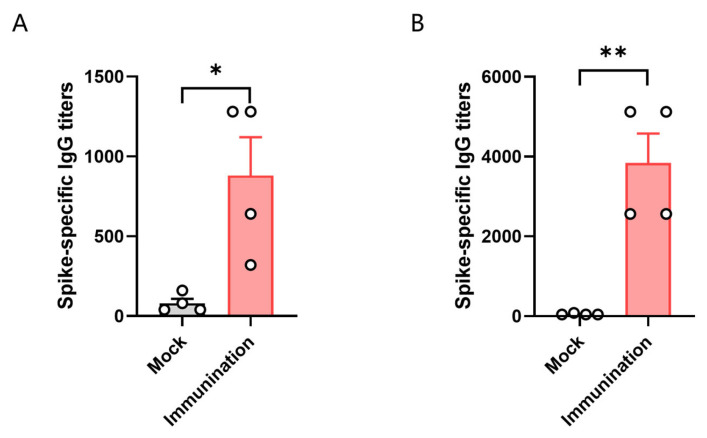
The titer of specific antibodies in the serum of HLA-A11/DR1 mice 14 days post-vaccination with inactivated SARS-CoV-2 (**A**). The titer of specific antibodies in the serum of C57BL/6 mice 14 days after vaccination with inactivated SARS-CoV-2 (**B**). * *p* < 0.05, ** *p* < 0.01.

To test the immune response of HLA-A11/DR1 transgenic mice, we divided HLA-A11/DR1 mice and C57BL/6 mice into two groups, with four mice in each group. Each mouse was immunized with 3 × 10^3^ TCID_50_ of inactivated SARS-CoV-2 virus. We then tested the antibody production in the serum of the mice 14 days after immunization with the inactivated virus. The results showed that specific IgG antibodies were induced in vivo in both HLA-A11/DR1 mice and C57BL/6 mice after immunization with the inactivated virus. The results showed that the antibody levels produced by C57BL/6 mice were approximately 4.3 times higher than that of HLA-A11/DR1 mice (Figure 3). The detection of serum antibodies indicated that HLA-A11/DR1 mice have a normal immune response.

### 3.4. Screening for HLA-Restricted Positive Epitope Peptides

**Figure 4:**
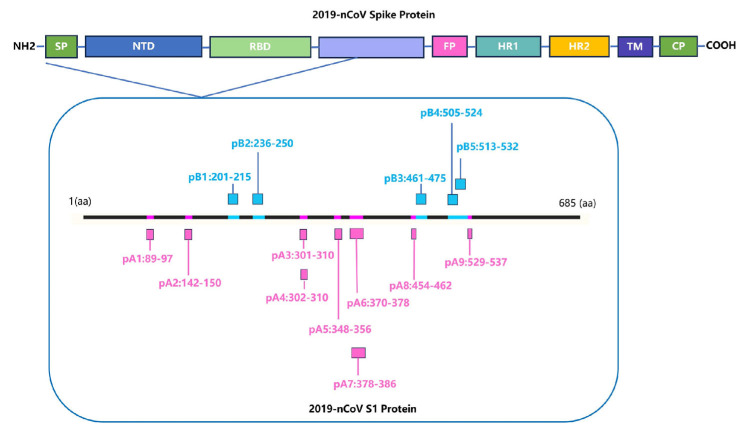
Screening for HLA-restricted positive epitope peptides. We selected 14 HLA-restricted epitope peptides from the novel coronavirus epitope library established by our research group, including nine HLA-A*11:01-restricted epitope peptides (represented in purple): pA1: GVYFASTEK [27,28,29], pA2: GVYYHKNNK [28,30], pA3: CTLKSFTVEK [28], pA4: TLKSFTVEK [30], pA5: ASVYAWNRK [30], pA6: NSASFSTFK [28], pA7: KCYGVSPTK [31], pA8: RLFRKSNLK [29], pA9: KSTNLVKNK [32]. It also included five HLA-DRB1*01:01-restricted epitope peptides (represented in blue): pB1: FKIYSKHTPINLVRD [33], pB2: TRFQTLLALHRSYLT [33,34], pB3: LKPFERDISTEIYQA [35], pB4: YQPYRVVVLSFELLHAPATV [36], pB5: LSFELLHAPATVCGPKKSTN [36].

Identifying HLA-restricted epitopes helps to screen for highly conserved epitopes and monitor the immunogenicity of epitopes. HLA-restricted epitopes are crucial for activating CD4+ and CD8+ T cells. Identifying the immunogenicity of these epitopes is beneficial for designing peptide vaccines that can induce T cell responses and can better cope with humororal immune escape caused by high-frequency mutation of viruses. We screened for HLA-A*11:01 and HLA-DRB1*01:01-restricted positive epitope peptides, which were conserved and experimentally verified using human samples. We identified nine HLA-A*11:01-restricted epitope peptides and five HLA-DRB1*01:01-restricted epitope peptides (Figure 4).

### 3.5. HLA-A11/DR1 Mice Are Capable of Producing a Normal Cellular Immune Response

IFN-γ, as a cytokine secreted by immune-active cells, plays a significant immunomodulatory role in inducing antiviral immunity. This includes the activation of cytotoxic T lymphocytes (CTLs), natural killer (NK) cells, and phagocytic cells, among others. After mice are immunized with inactivated virus, the body can produce IFN-γ. Detecting the production level of IFN-γ can indicate the cellular immune response triggered by peptide stimulation after the mouse is immunized with inactivated virus.

**Figure 5:**
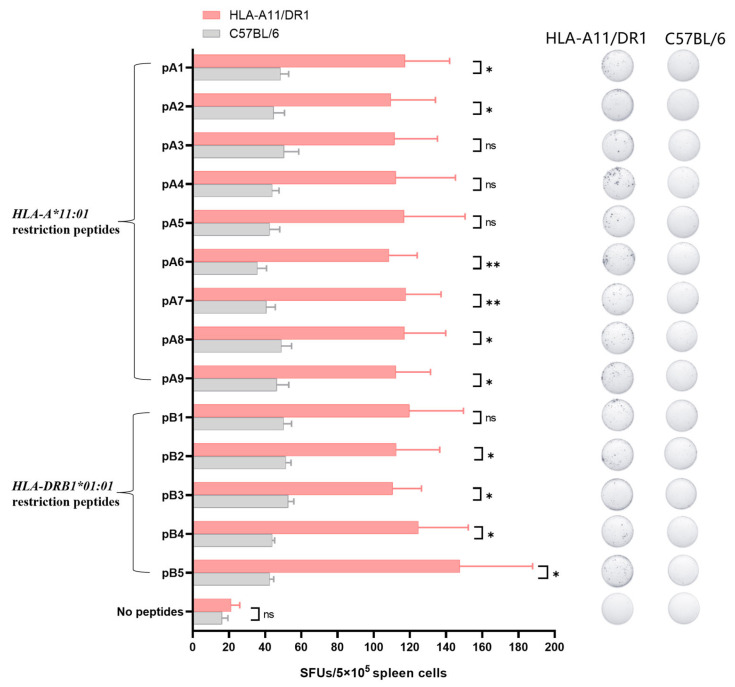
The cell-mediated immune response of HLA-A11/DR1 transgenic mice was tested using positive epitope peptides. Four HLA-A11/DR1 mice and four C57BL/6 mice were intramuscularly injected with inactivated SARS-CoV-2 virus. Fourteen days after vaccination, the mice were euthanized and their spleen tissues were collected. The spleen cells were stimulated with 14 selected HLA-restricted epitope peptides, and the production of IFN-γ in the spleen cells was detected by ELISpot. The study was analyzed by the Mabtech IRIS FluoroSpot/ELISpot reader (Apex 1.1), using RAWspot technology for multiplexing at the single-cell level. * *p* < 0.05, ** *p* < 0.01, “ns” means no significance.

We used the screened HLA-restricted epitope peptides to verify the T cell immune response in HLA-A11/DR1 mice. Fourteen days after immunization the mice with inactivated SARS-CoV-2 virus, we harvested their spleens, stimulated splenocytes with the selected peptides, and measured IFN-γ spot formation. The analysis was conducted using C57BL/6 mice and an unstimulated group as controls. The results showed that HLA-A11/DR1 mice exhibited increased IFN-γ production than C57BL/6 mice (Figure 5 and Figure S1), indicating that HLA-A11/DR1 mice generate a physiological cellular immune response when stimulated with HLA-restricted epitope peptides.

## 4. Discussion

Since its outbreak in 2019, SARS-CoV-2 has continued to spread among humans, posing a persistent threat to global health. During SARS-CoV-2 infection, the coronavirus spike (S) glycoprotein facilitates the entry of SARS-CoV-2 into host cells through the host receptor ACE2 [2,37,38,39]. Researchers have studied mouse models that can reflect the disease progression and pathogenic characteristics after human infection with SARS-CoV-2 [25,26,40,41]. However, there are interspecific differences between the immune systems of mice and humans, which prevent the mouse model from reflecting the true immune response status in humans. Currently, various mouse models are being used for the study of viral pathogenic mechanisms and vaccines [42,43]. In this paper, we used a double-transgenic humanized mouse model HLA-A11/DR1. It is worth noting that this transgenic mouse has removed the endogenous mouse MHC. The MHC of mice is called H-2. Silencing of the H-2 gene was detected in mice, rendering the endogenous H-2 of the transgenic mice non-functional. However, the mice can stably express HLA-A*11:01 and HLA-DRB1*01:01 molecules, resulting in the mouse immune system being regulated solely by HLA and thus enabling the simulation of human immune response status [23].

In this study, we used humanized HLA-A11/DR1 transgenic mice, which were modified based on C57BL/6 mice for MHC humanization. The research showed that C57BL/6 mice cannot enable the virus to replicate effectively in vivo. However, after all, there are certain differences between HLA-A11/DR1 mice and wild-type mice. Therefore, the pathogenic characteristics of HLA-A11/DR1 mice are worth exploring. In the study, we set up the experiment of HLA-A11/DR1 transgenic mice infected with SARS-CoV-2. By observing the weight changes in mice, we found that neither HLA-A11/DR1 mice nor wild-type mice showed significant weight changes. Next, we also tested the viral load in the lung tissues of the mice. Compared with hACE2 mice that can replicate the virus in vivo, the viral content detected in the lungs of HLA-A11/DR1 mice was very low, and the distribution of viral antigens was hardly detected. The results indicated that HLA-A11/DR1 mice could not replicate the virus in vivo. We speculate that this may be because mice do not have the hACE2-specific receptor, as studies have shown that hACE2 is the receptor for SARS-CoV-2 to invade host cells. To verify whether HLA-A11/DR1 mice have normal immune functions, an inactivated SARS-CoV-2 virus was administered. Antibodies were detected in the serum 14 days after immunization. The results showed that C57BL/6 mice produced higher antibodies. This might be because the HLA-A11/DR1 mice we used lack β2m, thus lacking functional MHC class I and class I-like molecules, including FcRn, leading to increased metabolism of antibodies and resulting in a decrease in antibody levels in the test mice [44].

The novel coronavirus enters host cells by binding to human angiotensin-converting enzyme 2 (ACE2) via the SARS-CoV-2 spike protein, which is targeted by neutralizing antibodies produced by vaccine-induced immunity. These antibodies can reduce the severity of viral infections [45,46]. However, as the virus mutates, antibody neutralization weakens, resulting in humoral immune escape [47]. Vaccines targeting T cell epitopes can directly eliminate infected cells. As conservative antigen epitopes are selected for vaccine design, cellular immunity induced by these vaccines is less likely to be affected by mutations in the SARS-CoV-2 spike protein. Thus, designing T cell epitope-based vaccines is crucial [48]. In previous studies, people screened and analyzed epitopes through human PBMCs to identify the immunogenicity of epitopes. Methods for screening and validating antigenic epitopes include using epitope databases, constructing cell lines, and validating with clinical samples [28,49]. Validation with clinical samples accurately simulates the human immune environment, for assessing the immunogenicity and immune protection effects of candidate antigenic epitopes in hosts. However, due to the challenges in obtaining clinical samples, timely and extensive experimentation is not feasible. Studies have shown that the HLA-A11/DR1 mouse model can identify the immunogenicity of epitopes. Therefore, it can be directly used for screening and identifying new epitopes, thereby obtaining the information about the immunogenicity of epitopes in a timely manner. In this study, we screened the HLA-A*11:01 and HLA-DRB1*01:01 restricted positive epitopes reported in the literature, and verified by using human samples to study the cellular immune response of HLA-A11/DR1 transgenic mice. Fourteen days after immunization with the inactivated virus, mouse spleen cells were stimulated with the selected positive peptides. Compared with wild-type mice, spleen cells from HLA-A11/DR1 mice produced more IFN-γ. Wild-type mice also stimulated a portion of IFN-γ, which may be due to a certain cross-reaction among epitopes. Although C57BL/6 mice do not have HLA, the structure and function of mouse and human MHC are very similar, which may lead to the overlapping recognition of certain peptides, and mouse T cells may recognize HLA-restricted peptides through cross-reactivity. Although C57BL/6 mice also produced some IFN-gamma, the amount of IFN-gamma produced by HLA-A11/DR1 mice was higher than that of C57BL/6 mice, confirming that HLA-A11/DR1 mice indeed produced specific spots. The epitopes we screened are immunogenic epitope peptides verified by experiments such as intracellular staining (ICS) and MHC tetramer staining using cells isolated from human PBMC samples. When we stimulated the spleen cells of HLA-A11/DR1 mice immunized with inactivated virus with these epitope peptides, specific spots were produced compared with the unstimulated group and C57BL/6 mice. Therefore, we conclude that HLA-A11/DR1 mice can also identify the immunogenicity of epitopes, and can serve as an important tool for screening and verifying the immunogenicity of epitopes instead of PBMC. The research shows that although HLA-A11/DR1 transgenic mice cannot enable the virus to replicate, they are able to produce a normal immune response. They have the same effect as the epitopes verified using human PBMCs in previous studies. Therefore, HLA-A11/DR1 transgenic mice can provide an important research tool and technical platform for the screening of T cell epitopes. Identifying HLA-restricted epitopes helps to screen for highly conserved epitopes and monitor the immunogenicity of epitopes, and allow the selected epitopes to be further used in vaccine design. Currently, many people have further constructed multi-epitope peptide vaccines with the screened epitopes or designed vaccines after tandem connection of peptides [50,51].

HLA is polymorphic, with many different alleles, and there are significant differences between different races and regions. Based on the HLA-A11/DR1 transgenic mouse model, we can consider constructing transgenic mouse models that cover more HLA-restricted epitopes, which can then be used to screen epitopes with different restrictions. In the next step of our research, we will mate HLA-A11/DR1 transgenic mice with mice that have receptors for SARS-CoV-2 infection. This will enable us to study the relationship between HLA and the virus pathogenic mechanism, as well as analyze the immunogenicity and protective efficacy of vaccines.

## 5. Conclusions

Overall, in this study, we verified, through immunization of inactivated viruses in mice, that HLA-A11/DR1 transgenic mice can be used to screen for epitopes with effects equivalent to those selected using human PBMCs. Therefore, in emergency situations, HLA-A11/DR1 mice can be used instead of PBMCs for the screening and identification of epitopes. There is no need to wait for patients to appear after the outbreak of SARS-CoV-2 before conducting experiments, thus enabling timely acquisition of information on the immunogenicity of epitopes. HLA-A11/DR1 mice can serve as a strategy for SARS-CoV-2 T-cell epitope screening. HLA-A11/DR1 mice can be used for the preliminary screening and analysis of new epitopes predicted from databases. This reduces the range of epitopes that need further verification and provides a prerequisite for the research on T-cell epitope vaccines.

## Data Availability

The data presented in this paper are available on request from the corresponding author.

## Supplementary Materials

The following supporting information can be downloaded at: https://www.mdpi.com/article/10.3390/vaccines13040416/s1, Figure S1: The ELISpot assay was used to detect the IFN-gamma produced by splenocytes in mice after immunization with inactivated virus.
