# Supplementary material for: Humanized Major Histocompatibility Complex Transgenic Mouse Model Can Play a Potent Role in SARS-CoV-2 Human Leukocyte Antigen-Restricted T Cell Epitope Screening
Source: Vaccines (Basel). 2025 Apr 15;13(4):416. doi: 10.3390/vaccines13040416 (PMC12031200; doi:10.3390/vaccines13040416)
Supplement: Supplementary file 1 [file vaccines-13-00416-s001.zip › vaccines-3468548-supplementary.docx]

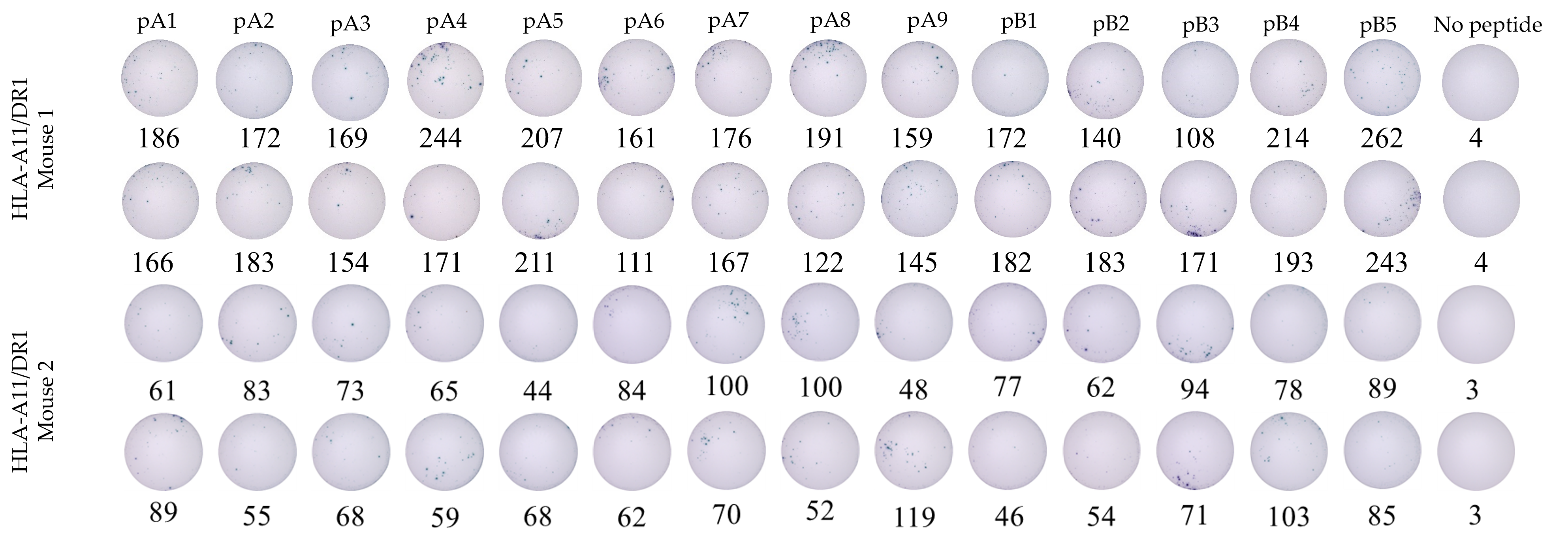

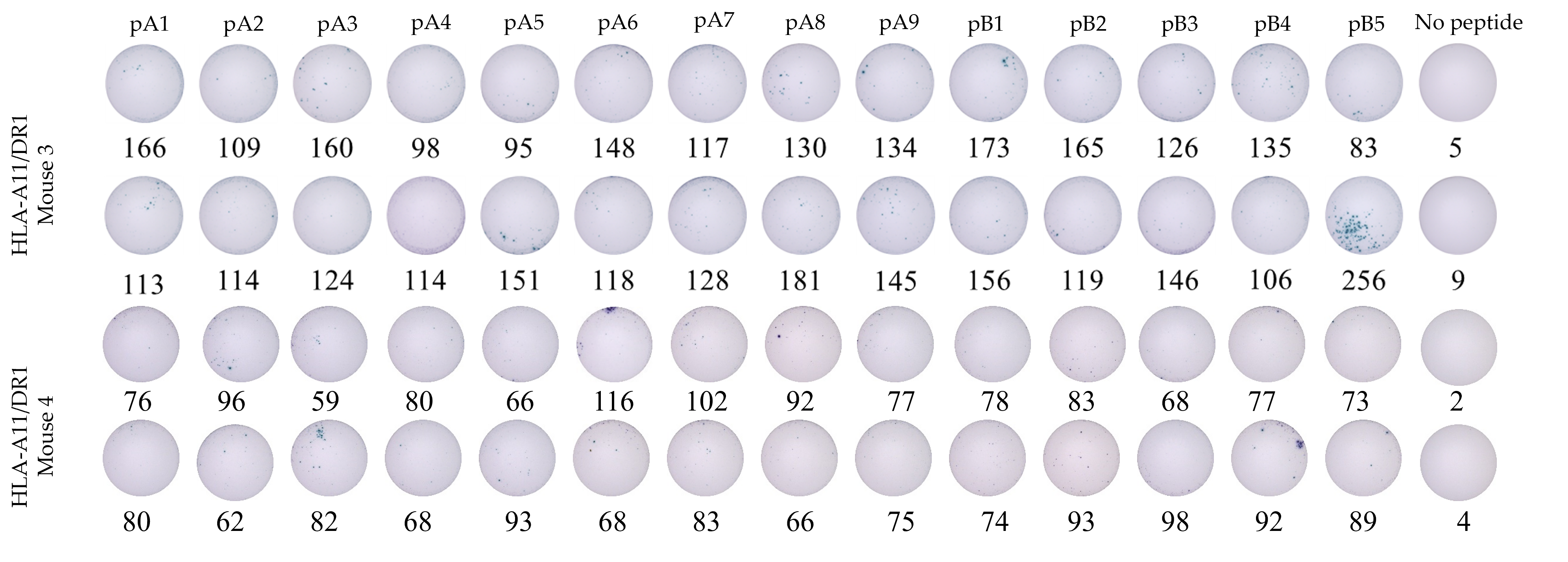

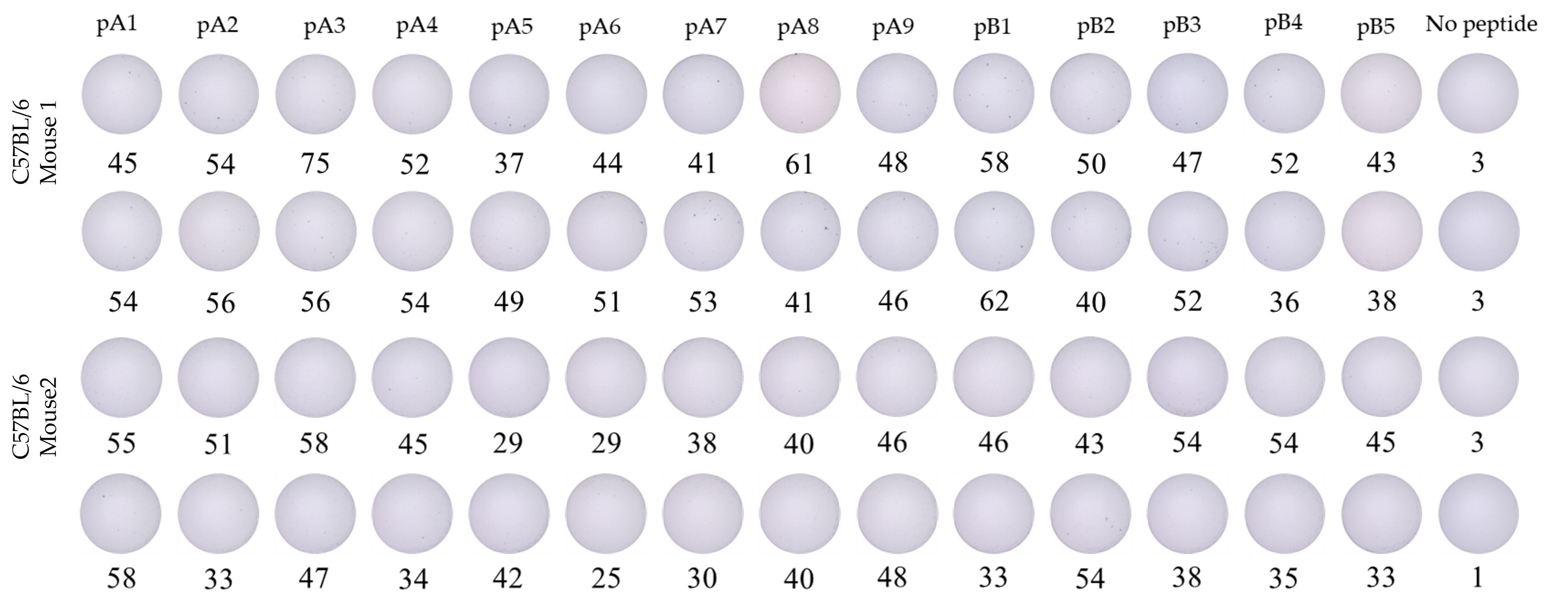

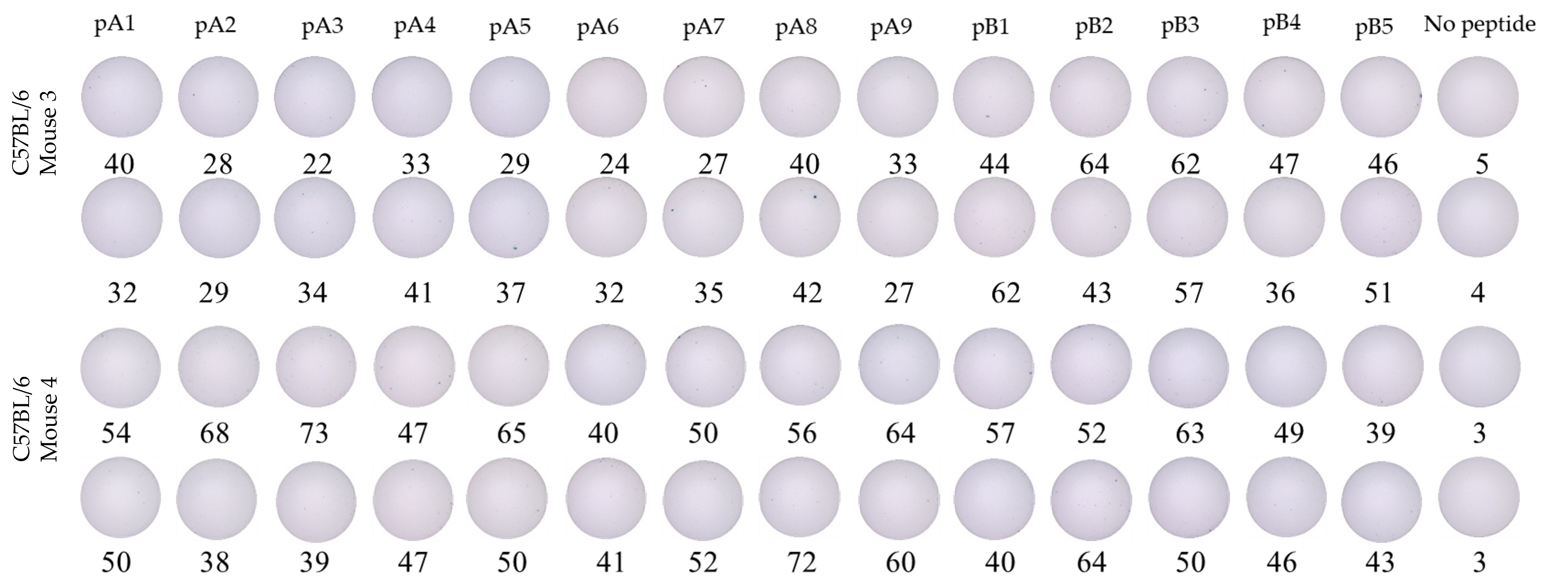


**Figure S1:** The ELISpot assay was used to detect the IFN-gamma produced by splenocytes in mice after immunization with inactivated virus. Fourteen days after mouse immunization with inactivated virus, splenocytes were collected from the mice, and the ELISpot assay was used to detect the production of IFN-gamma using the screened epitope peptides, in which 5×10^5^ cells were in the peptide-stimulated group and 1×10^5^ cells in the unstimulated group.
